# Low Nephron Number Induced by Maternal Protein Restriction Is Prevented by Nicotinamide Riboside Supplementation Depending on Sirtuin 3 Activation

**DOI:** 10.3390/cells11203316

**Published:** 2022-10-21

**Authors:** Anna Pezzotta, Luca Perico, Marina Morigi, Daniela Corna, Monica Locatelli, Carlamaria Zoja, Ariela Benigni, Giuseppe Remuzzi, Barbara Imberti

**Affiliations:** Istituto di Ricerche Farmacologiche Mario Negri IRCCS, Centro Anna Maria Astori, Science and Technology Park Kilometro Rosso, 24126 Bergamo, Italy

**Keywords:** renal development, fetal programming, glomerular number, low protein diet, sirtuin 3, mitochondria, nicotinamide riboside

## Abstract

A reduced nephron number at birth, due to critical gestational conditions, including maternal malnutrition, is associated with the risk of developing hypertension and chronic kidney disease in adulthood. No interventions are currently available to augment nephron number. We have recently shown that sirtuin 3 (SIRT3) has an important role in dictating proper nephron endowment. The present study explored whether SIRT3 stimulation, by means of supplementation with nicotinamide riboside (NR), a precursor of the SIRT3 co-substrate nicotinamide adenine dinucleotide (NAD^+^), was able to improve nephron number in a murine model of a low protein (LP) diet. Our findings show that reduced nephron number in newborn mice (day 1) born to mothers fed a LP diet was associated with impaired renal SIRT3 expression, which was restored through supplementation with NR. Glomerular podocyte density, as well as the rarefaction of renal capillaries, also improved through NR administration. In mechanistic terms, the restoration of SIRT3 expression through NR was mediated by the induction of proliferator-activated receptor γ (PPARγ) coactivator-1α (PGC-1α). Moreover, NR restored SIRT3 activity, as shown by the reduction of the acetylation of optic atrophy 1 (OPA1) and superoxide dismutase 2 (SOD2), which resulted in improved mitochondrial morphology and protection against oxidative damage in mice born to mothers fed the LP diet. Our results provide evidence that it is feasible to prevent nephron mass shortage at birth through SIRT3 boosting during nephrogenesis, thus providing a therapeutic option to possibly limit the long-term sequelae of reduced nephron number in adulthood.

## 1. Introduction

Disturbances of the intrauterine environment negatively impact normal embryonic development in animals and humans [1]. During pregnancy, maternal malnutrition is among those conditions that have the most harmful effects on embryonic development, leading to intrauterine growth retardation and low birth weight, both of which significantly contribute to abnormal programming of the kidneys and a reduced number of nephrons [2,3,4,5]. Evidence that has accumulated over the last three decades suggests that renal diseases start in utero [4,5]. Brenner and colleagues first proposed the developmental origins of kidney disease; low nephron number is one of the main contributors to chronic kidney disease (CKD) susceptibility in adulthood [4,6,7]. In line with this hypothesis, several epidemiologic studies across multiple homogeneous populations have clearly demonstrated the association between low birth weight, low nephron endowment and a propensity toward CKD in later life [8,9,10]. Therefore, adverse developmental programming may have long-term and permanent consequences on the health of adult subjects, who are strongly predisposed to develop hypertension, cardiovascular diseases and CKD [11].

The molecular mechanisms that regulate nephron number are complex and not fully understood yet [12]. We recently found that in mice, kidney development relies on the activity of the mitochondrial protein sirtuin 3 (SIRT3) [13], which is a deacetylase whose activity is dependent on nicotinamide adenine dinucleotide (NAD^+^) availability [14,15,16]. Mice lacking SIRT3 exhibited reduced ureteric bud branching, a lower number of the sine oculis-related homeobox 2 (SIX2) positive progenitor cells, and impaired cell proliferation [13]. Overall, SIRT3 deficient mice experienced impaired nephrogenesis and a shortage of nephrons at birth, which is permanent [13]. Additionally, lack of SIRT3 in these mice leads to increased acute renal injury susceptibility and premature death in adulthood [17,18].

Similarly, a nephron deficit at birth has been established successfully in mice with gestational protein restriction, an animal model for intrauterine growth retardation that is useful for investigating the mechanisms underlying the developmental changes in the kidney that occur during malnutrition [19]. In this setting, the offspring of mothers fed a low protein (LP) diet had a reduced number of nephrons and morphological and ultrastructural alterations in the glomerular architecture [20]. A recent paper showed that podocyte number can be developmentally programmed and was lower in rats born to mothers fed a LP diet [21]. Moreover, a LP diet during pregnancy induces significant changes in the gene expression of the developing kidney, the metanephroi [22,23].

Based on these premises, the main aim of the present study was to investigate whether in a murine model of low nephron number induced by a maternal LP diet, a pharmacological strategy based on SIRT3 targeting could support nephron development and prevent nephron loss. To this end, we chose to supplement pregnant mice fed a LP diet with the nicotinamide riboside (NR) a precursor of NAD^+^, which is the co-substrate of SIRT3 enzymatic activity [16,24], as well as a regulator of SIRT3 expression [25].

Here, we show that a nephron number deficit and impaired renal architecture of mice born to mothers fed a LP diet can be restored by NR supplementation during pregnancy through the induction of SIRT3 expression and activity. Specifically, gestational NR supplementation during pregnancy restores SIRT3 deacetylase activity and mitochondrial wellness in the offspring, leading to the normalization of nephrogenesis.

## 2. Materials and Methods

### 2.1. Animal Experiments

All procedures involving animals were performed in accordance with institutional guidelines in compliance with national (D.L.n.26, 4 March 2014), and international laws and policies (directive 2010/63/EU on the protection of animals used for scientific purposes). This study was approved by the Institutional Animal Care and Use Committees of Istituto di Ricerche Farmacologiche Mario Negri IRCCS and by the Italian Ministry of Health (approval number 16/2017-PR). This study was carried out in compliance with the ARRIVE guidelines [26].

Seven-week-old female and male C57BL/6 mice were purchased from Charles River Laboratories Italia (Calco, Lecco, Italy) and maintained in a pathogen-free facility at a constant temperature with a 12:12-h light-dark cycle. A total of *n* = 36 females and *n* = 18 males were used for matings. At matings, mice were randomly allocated to three different groups: (1) standard diet (SD), (2) isocaloric LP diet, and (3) isocaloric LP diet supplemented with NR (LP + NR). The chow used for the SD consisted of 18.6% protein, 44.2% carbohydrates, and 6.2% fat (2018S, Envigo, Indianapolis, IN, USA). The LP diet composition was 6.1% protein, 75.6% carbohydrates, and 5.5% fat (TD.90016, Envigo). Nicotinamide riboside (Niagen^®^) was provided by ChromaDex, Inc. (Irvine, CA, USA) and was given to mice daily in drinking water at a concentration of 0.36 g/kg/day. Mice had free access to chow and drinking water. No changes in water or food intake were observed in experimental groups. A total of *n* = 5 pregnant mice per group were obtained. No fetal mortality occurred in any of the experimental groups. At day 1, both male and female pups were sacrificed and kidneys were collected and processed for subsequent analysis. No inclusion or exclusion parameters were used in our studies. Investigators were not blinded to treatments, but no subjective assessments were made.

In selected experiments, 8-week-old *Sirt3^−/−^* female and male mice, generated in a mixed genetic background (provided by Professor Frederick Alt, Harvard Medical School, Boston, MA, USA) were used [13,18,27]. At mating, mice were randomly allocated to the experimental groups: (1) SD, and (2) SD + NR. As a control, their C57BL/6x129 wild-type (WT) littermates were used. A total of *n* = 7 matings were performed to obtain a sample size of *n* = 3 pregnant mice per group. As above, both male and female pups were sacrificed at day 1 and their kidneys were collected and processed for subsequent analysis.

### 2.2. Estimation of Glomerular Number

Maceration of the whole kidney was performed with HCl as previously described [13]. Briefly, isolated kidneys were incubated in NH_3_ for 2 h, then incubated in 6N HCl at 37 °C in 2.5 mL. Pipetting up and down after maceration further disrupted the kidneys. Distilled water (7.5 mL) was added to the sample, followed by incubation at 4 °C overnight. Then, 100 μL of macerate was pipetted into a cell culture dish with a grid and the number of glomeruli per area was counted.

### 2.3. Glomerular Podocyte Count

Formalin-fixed, 3-μm paraffin-embedded kidney sections were incubated with Peroxidazed 1 (PX968H, Biocare Medical, Pacheco, CA, USA), after antigen retrieval in a decloaking chamber with Rodent decloaker (RD913M, Biocare Medical) buffer. After blocking for 30 min with Rodent Block M (RBM961G, Biocare Medical), sections were incubated with rabbit anti- Wilms Tumor 1 (WT1, 1:600; ab89901, abcam, Cambridge, UK) antibody followed by Rabbit on Rodent horseradish peroxidase (HRP)-Polymer (RMR622G, Biocare Medical,) for 30 min at room temperature. Staining was visualized using diaminobenzidine (BDB2004H, Biocare Medical) substrate solutions. Slides were counterstained with Mayer’s hematoxylin (MHS80-2.5L, Bio Optica, Milan, Italy), mounted with Eukitt mounting medium (09-00250, Bio Optica) and finally observed using light microscopy (ApoTome, Axio Imager Z2, Zeiss, Oberkochen, Germany). Negative controls were obtained by omitting the primary antibody on adjacent sections. At least 15 glomeruli/section for each animal were randomly acquired. The average number of podocytes per glomerulus and the glomerular volume were estimated using morphometric analysis, as previously described [28].

### 2.4. Immunoperoxidase Analysis

Formalin-fixed, 3-μm paraffin-embedded kidney sections were incubated with Peroxidazed 1 to quench endogenous peroxidase, after antigen retrieval in a decloaking chamber with Rodent decloaker buffer. After blocking for 30 min with Rodent Block M, sections were incubated with rabbit anti-Nitrotyrosine (1:100; 06-284, Merck Millipore, Burlington, MA, USA) or rabbit anti-CD31 (1:50; ab28364, abcam) antibody followed by Rabbit on Rodent HRP-Polymer for 30 min at room temperature. Stainings were visualized using diaminobenzidine substrate solutions. Slides were counterstained with Mayer’s hematoxylin, mounted with Eukitt mounting medium and finally observed using light microscopy (ApoTome, Axio Imager Z2). Negative controls were obtained by omitting the primary antibody on adjacent sections. At least 20 non overlapping fields for each section were examined. For CD31 staining, images were analyzed using ImageJ 1.40 g software. Digitized images were dichotomized using a threshold for staining, and the values were expressed as the percentage of staining per glomerulus or per total area of the acquired field, as appropriate.

### 2.5. Immunofluorescence Analysis of Renal Cell Proliferation

For the immunofluorescence analysis of kidney sections, 3-μm periodate-lysine paraformaldehyde (PLP)-fixed cryosections were air dried. To detect phospho-Histone H3 antibody (pHH3), antigen retrieval was performed in citrate buffer 10 mmol/L (pH 6.0) at boiling temperature for 20 min, followed by incubation with citrate buffer (20 min) at room temperature to enhance the reactivity of antibodies to antigens. Slides were washed with PBS 1× and incubated with 1% BSA to block nonspecific sites. Rabbit anti-pHH3 (1:75; #9701, Cell Signaling, Danvers, MA, USA) was used followed by the specific Cy3-conjugated secondary antibody (Jackson ImmunoResearch Laboratories, Cambridge, UK). Nuclei were stained with 4′,6-Diamidino-2-phenylindole dihydrochloride (DAPI, 28718-90-3, Sigma-Aldrich, St. Louis, MO, USA) and the renal structure with fluorescein wheat germ agglutinin (WGA; FL-1021, Vector Laboratories, Burlingame, CA, USA). Finally, slides were mounted using Dako Fluorescence Mounting Medium (S3023, Agilent Technologies, Santa Clara, CA, USA) and examined with an inverted confocal laser scanning microscope (Leica TCS SP8, Leica Microsystems, Wetzlar, Germany). Negative controls were obtained by omitting primary antibodies on adjacent sections. pHH3-positive cells in kidney tissue were evaluated in at least 10 HPF/section (*n* = 3 mice for each group).

### 2.6. Protein Extraction and Western Blot Analysis

Kidneys were isolated at day 1 and homogenised in CelLytic MT (C3228, Sigma-Aldrich), supplemented with a protease inhibitor cocktail (P8340, Sigma-Aldrich). Each sample consisted of a pool of at least 4 isolated kidneys. Following centrifugation at 16,000× *g* for 10 min at 4 °C, lysates were collected and total protein concentration was determined using DC™ assay (5000112, Bio-Rad Laboratories, Hercules, CA, USA).

Equal amounts of total proteins (30 μg) were separated on 12% SDS-PAGE under reducing conditions and transferred to nitrocellulose membranes (1704159, Bio-Rad Laboratories). After blocking with 5% bovine serum albumin (BSA A7030, Sigma-Aldrich) in Tris-buffered saline (TBS) supplemented with 0.1% Tween-20 (P1379, Sigma-Aldrich), membranes were incubated overnight at 4 °C with the following antibodies: goat anti-SIRT3 (1:1000; ab118334, abcam), proliferator-activated receptor γ (PPARγ) coactivator-1α (PGC-1α; 1:1000; ab54481, abcam), sheep anti-superoxide dismutase 2 (SOD2; 1:1000; 574596, Merck Millipore), rabbit anti-SOD2 acetyl lysine 68 (SOD2^KAc68^; 1:1000; ab137037, abcam), mouse anti-optic atrophy 1 (OPA1; 1:1000; 612606, BD Bioscience, Allschwil, Switzerland), rabbit anti-pan acetyl lysine (1:1000; PTM-105, ptmbiolabs, Chicago, Il, USA). Mouse anti-α-tubulin (1:2000; T9026, Sigma-Aldrich) was used as the sample-loading control in total kidney extracts.

The signals were visualized on an Odyssey^®^FC Imaging System (LiCor, Lincoln, NE, USA) with infrared (IR) fluorescence using a secondary goat anti-rabbit IRDye 680LT antibody (1:1000; FE3680210, LiCor) and a goat anti-mouse IRDye 800CW (1:1000; FE30926210, LiCor) or with an enhanced chemiluminescence-Western Blotting Detection Reagent (Pierce, ThermoFisher, Waltham, MA, USA) using donkey anti-goat horseradish peroxidase (HRP)-conjugated secondary antibodies (1:20,000; AP180P, Sigma-Aldrich), as appropriate.

Bands were quantified with densitometry using the Image Studio Lite 5.0 (LiCor) software. SOD2 acetylation was expressed as the ratio between the band of SOD2^KAc68^ and total SOD2, while OPA1 acetylation was evaluated as the ratio between the band of acetylated-lysine that co-localized with the band corresponding to OPA1. 

All uncropped gels of representative Western Blots reported the main figures are shown in Figure S1.

### 2.7. Ultrastructural Analysis

Mitochondrial morphology was observed using transmission electron microscopy (TEM), as performed previously [29]. Fragments of kidney tissue were fixed overnight in 2.5% glutaraldehyde (340855, Sigma-Aldrich) in 0.1 M cacodylate buffer pH 7.4 (11652, Electron Microscopy Sciences, Hatfield, PA, USA) and washed repeatedly in the same buffer. After post-fixation in 1% OsO_4_, specimens were dehydrated through ascending grades of alcohol and embedded in Epon resin. Ultrathin sections were stained with uranyl acetate replacement (UAR; 22405, Electron Microscopy Sciences) and lead citrate (22410, Electron Microscopy Sciences,) and examined using transmission electron microscopy (Fei Morgagni 268D, Philips, Hillsboro, OR, USA). Quantification of altered mitochondria was estimated on digitised EM pictures at 11,000 × and expressed as the number of altered mitochondria out of total mitochondrial number (%) in proximal tubules. The analysis was performed in *n* = 6 individual tubules in *n* = 3 kidneys from 3 newborn mice per group.

### 2.8. Statistical Analysis

Results were expressed as mean ± standard error of the mean (SEM). Data analysis was performed using Graph Pad Prism software (Graph Pad, San Diego, CA, USA). The sample size for each analysis is indicated in the corresponding Figure legend. Comparisons were made using one-way ANOVA with Tukey’s multiple comparisons post hoc test, and the statistical significance was defined as a *p*-value < 0.05.

## 3. Results

### 3.1. NR Supplementation Restores Low Nephron Number and Renal SIRT3 Expression in Maternal LP Diet Offspring

**Figure 1:**
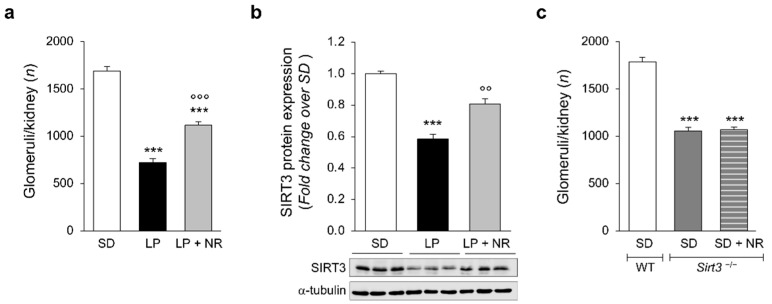
NR supplementation restores nephron number and SIRT3 expression in the kidneys of maternal LP diet offspring. (**a**) Glomerular number quantified in the kidney of newborn mice (day 1) born to mothers fed a SD during pregnancy or a LP diet supplemented or not with NR; *n* = 14 kidneys from 14 newborn mice for each group. (**b**) Representative Western blots and densitometric analysis of SIRT3 protein expression in total kidney extracts harvested from newborn mice (day 1) from mothers fed with SD (*n* = 8 samples), LP diet alone (*n* = 9 samples) or with NR (*n* = 9 samples). Each sample consisted of a pool of at least 4 kidneys from 4 newborn mice. (**c**) Quantification of the glomerular number in kidneys of the offspring of WT (*n* = 12 kidneys from 12 newborn mice) or *Sirt3^−/−^* mice fed a SD supplemented or not with NR during pregnancy (*n* = 11 and *n* = 14 kidneys from 11 and 14 newborn mice, respectively). Results are presented as mean ± SEM and were analyzed with ANOVA with Tukey’s post hoc test. *** *p* < 0.001 vs. SD; °° *p* < 0.01, and °°° *p* < 0.001 vs. LP.

Experimental studies show that restricted maternal protein intake impairs fetal renal development [20]. Here, we set up a murine model of a low nephron number in mice born to mothers that have been fed a LP diet between the time of mating and delivery. Prenatal consumption of LP significantly reduced the average body weight of the offspring at birth (day 1) as well as kidney weight and the kidney to body ratio (Table 1). In this setting, we performed renal tissue dissociation to quantify the number of glomeruli, from which the total nephron number in the kidneys can be extrapolated. Exposing pregnant mice to a LP diet resulted in the nephron endowment in offspring being significantly impaired (Figure 1a). The glomerular number observed at birth in mice born to mothers fed LP was 57% lower than in pups born to mothers that received a SD (Figure 1a).

Next, we evaluated whether mice born to mothers fed the LP diet had altered SIRT3 expression and activity in the kidneys. We first analyzed SIRT3 protein expression with Western Blot. As shown in Figure 1b, we found that at day 1 mice born to LP-fed mothers had significantly lower levels of SIRT3 in the kidney compared with offspring from SD-fed mothers. To modulate SIRT3, pregnant mice fed LP received a nicotinamide riboside (NR) supplement that resulted into a significant increase in renal SIRT3 protein expression (Figure 1b). Notably, when LP diet-fed pregnant mice were administered NR, the treatment attenuated nephron loss in the offspring and, indeed, the glomerular number was significantly higher than in mice that received only LP (Figure 1a).

The ability of NR to rescue nephron number during development was tested in mice that lacked SIRT3. Pregnant *Sirt3^−/−^* mice were fed a SD supplemented with NR. As shown in Figure 1c, NR supplementation failed to restore nephron numbers in *Sirt3^−/−^* newborns. These data indicate that NR effectively normalizes nephron endowment only in *Sirt3* competent mice.

### 3.2. NR Supplementation Improves Kidney Weight of Maternal LP Diet Offspring

Based on our finding—that maternal protein restriction resulted in a lower kidney and body weight at birth in newborn mice at day 1—as shown in Table 1, we investigated whether supplementing the maternal diet with NR during pregnancy could improve these parameters. We found that the number of mouse pups was not affected in any experimental group (Table 1). However, the body weight of pups born to mothers fed LP diet was lower than in the SD group but was not modulated by NR treatment (Table 1). Notably, the reduction in kidney weight induced by the LP diet was attenuated significantly by NR supplementation (Table 1).

### 3.3. NR Supplementation Normalizes Podocyte Density and the Renal Capillary Deficit in Maternal LP Diet Offspring

**Figure 2:**
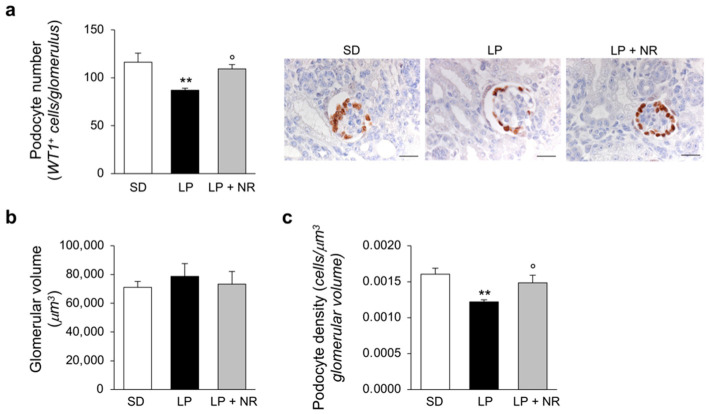
NR supplementation restores podocyte density in maternal LP diet offspring. (**a**) Quantification of podocyte number per glomerulus in maternal SD, LP or LP + NR offspring (*n* = 4 kidneys from 4 newborn mice per group) and representative images of renal tissues immunohistochemically stained for the podocyte marker WT-1. Scale bars, 20 μm. (**b**) Kidneys from newborn mice born to mothers fed a SD, LP or LP + NR were analyzed to quantify the glomerular volume (*n* = 4 kidneys from 4 newborn mice per group) and (**c**) podocyte density (*n* = 4 kidneys from 4 newborn mice per group). Results are presented as mean ± SEM and were analyzed with ANOVA with Tukey’s post hoc test. ** *p* < 0.01 vs. SD; ° *p* < 0.05 vs. LP.

The evidence of an impaired nephron number in LP diet offspring prompted us to investigate whether major glomerular cell populations, including podocytes and endothelial cells, which strongly impact and regulate renal function, are altered in mice born to mothers fed a LP diet and are affected by NR supplementation. We analyzed renal sections stained with the podocyte marker WT-1 and observed that maternal protein restriction during pregnancy significantly reduced the number of podocytes per glomerulus in offspring (Figure 2a) which, given the unchanged glomerular volume (Figure 2b), resulted in decreased podocyte density (Figure 2c). NR administration to LP-mothers had a rescuing effect on podocyte number and podocyte density, which were completely normalized and did not differ from those found in mice on the SD diet (Figure 2a,c).

**Figure 3:**
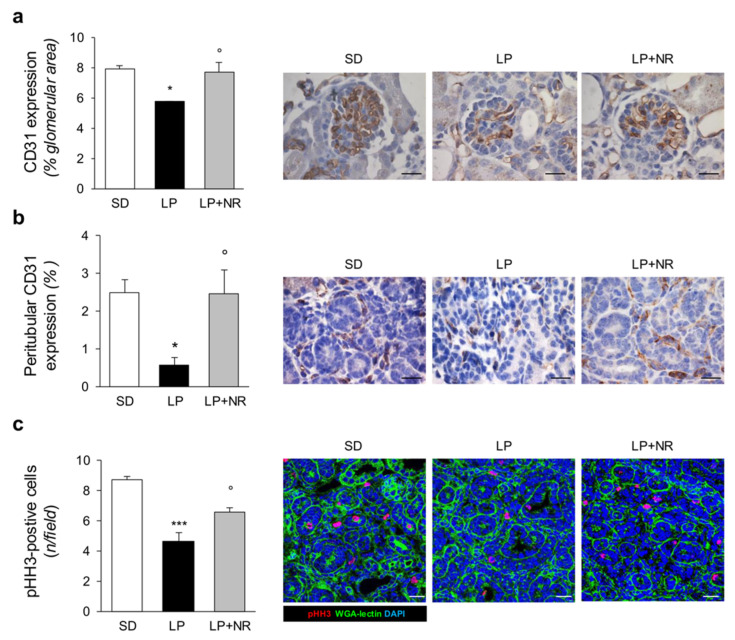
NR supplementation protects renal vasculature and increases cell proliferation in maternal LP diet offspring. (**a**) Quantification and representative images of the endothelial marker CD31 in glomeruli of SD, LP or LP + NR offspring (*n* = 3 kidneys from 3 newborn mice per group). Scale bars, 20 μm. (**b**) Quantification and representative images of the peritubular area positive for CD31 in SD, LP or LP + NR offspring (*n* = 3 kidneys from 3 newborn mice per group). Scale bars, 20 μm. (**c**) Representative images and quantification of cell proliferation assessed by pHH3 staining in kidneys from day 1 mice born to mothers fed a SD or LP supplemented or not with NR (*n* = 3 kidneys from 3 newborn mice per group). Scale bars, 20 μm. Results are presented as mean ± SEM and were analyzed with ANOVA with Tukey’s post hoc test. * *p* < 0.05, *** *p* < 0.001 vs. SD; ° *p* < 0.05 vs. LP.

Moreover, exposure to LP diet during pregnancy had a significant impact on renal tissue vascularization in newborn mice. Indeed, the analysis of the endothelial marker CD31 showed that glomeruli from mice born to LP-fed mothers had fewer glomerular capillaries (Figure 3a). An even greater effect of LP diet was observed in interstitial areas on peritubular capillary frequency (Figure 3b). NR supplementation to LP-fed mothers was able to completely restore glomerular capillary density and normalize peritubular capillary rarefaction in offspring (Figure 3a,b).

### 3.4. NR Supplementation Activates Renal Cell Proliferation in Maternal LP Diet Offspring

A reduced protein intake during pregnancy impacts cell proliferation during the embryonic development of the kidney [30]. We therefore studied cell proliferation in the kidneys of newborn mice born to LP-fed mothers that received NR by analyzing the expression of pHH3, a marker of the G2/M phase of the cell cycle. In SD-mice, we found discrete and widespread expression of pHH3-positive cells in renal tissue at day 1 (Figure 3c). The offspring of LP-fed mice exhibited a great reduction in the number of proliferating cells per field, which was significantly enhanced by NR, indicating that NR supplementation can restore cell cycle activity in the neonatal kidney (Figure 3c).

### 3.5. NR Supplementation Re-Establishes PGC-1α Expression in Maternal LP Diet Offspring

**Figure 4:**
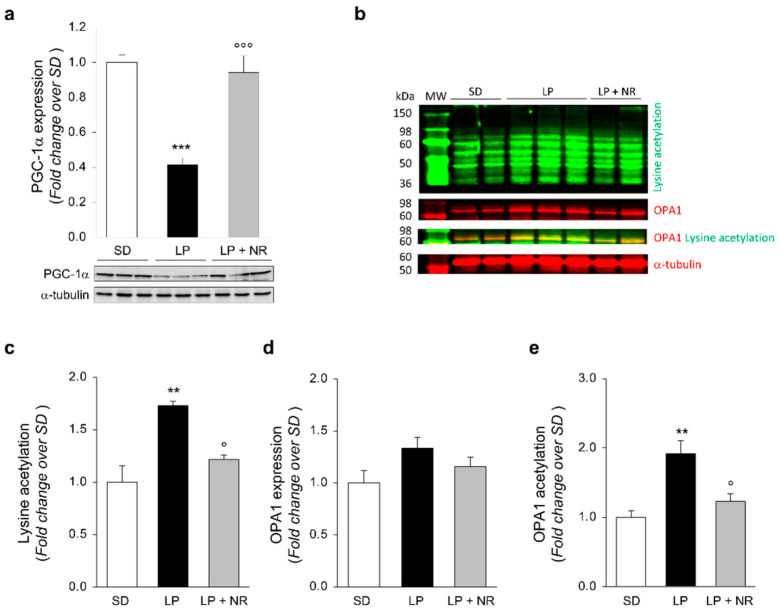
NR supplementation upregulates PGC-1α in maternal LP diet offspring and normalizes hyperacetylation of mitochondrial proteins. (**a**) Representative Western Blots and densitometric analysis of PGC-1α protein expression in total kidney extracts harvested from newborn mice (day 1) from mothers fed a SD, LP diet alone or with NR (*n* = 7 samples per group). Each sample consisted of a pool of at least 4 kidneys from 4 newborn mice. (**b**–**e**) Representative Western Blots (**b**) and densitometric analysis of lysine acetylation (**c**), OPA1 expression (**d**), and OPA1 acetylation (**e**) in total kidney extracts harvested from newborn mice (day 1) from mothers fed with SD, LP diet alone or with NR (*n* = 3 samples per group). Each sample consisted of a pool of at least 4 kidneys from 4 newborn mice. OPA1 acetylation has been evaluated as the colocalizing signal (yellow) between acetyl lysine (green) and OPA1 (red). In all gels, α-tubulin was used as a sample loading control. Results are presented as mean ± SEM and were analyzed with ANOVA with Tukey’s post hoc test. ** *p* < 0.01, and *** *p* < 0.001 vs. SD; ° *p* < 0.05, and °°° *p* < 0.001 vs. LP.

To investigate the molecular determinants through which NR could restore SIRT3 expression, we examined PGC-1α, which is a crucial modulator of mitochondrial function and induces *Sirt3* gene expression by binding to estrogen-related receptor elements mapped in the promoter region [25]. Western Blot analysis revealed that PGC-1α expression was significantly impaired in renal tissues from the offspring of LP-fed mothers and was significantly increased by NR treatment (Figure 4a). These data suggest that NR is able to boost SIRT3 expression in maternal LP diet mouse offspring by increasing PGC-1α expression.

### 3.6. NR Supplementation Reduces Hyperacetylation of the SIRT3 Target OPA1 in Maternal LP Diet Offspring

We asked ourselves whether NR could affect the deacetylase activity of SIRT3. Western Blot analysis with a specific antibody that detects protein acetylation at lysine residues in total renal extracts was performed. As shown in Figure 4b,c, the offspring of LP-fed mothers exhibited a significant increase in total acetylation levels compared to the offspring of SD-fed mothers. In newborn mice born to LP-fed mice that received NR supplement during pregnancy, we found a reduction in total protein acetylation on lysine residues (Figure 4b,c), possibly suggesting an increase in SIRT3 deacetylase activity.

Having identified an altered acetylome profile in newborn mice from LP-fed mothers, we sought to evaluate the acetylation status of different proteins whose activity is specifically regulated by SIRT3-dependent deacetylation. First, we investigated OPA1, a dynamin-like GTPase involved in mitochondrial inner membrane fusion [31,32]. While we found unchanged levels of total OPA1 protein expression in LP offspring, compared to the offspring of SD-fed mothers (Figure 4b,d), a significant increase in OPA1 lysine acetylation was found in kidneys from the offspring of LP-fed mothers and NR significantly reduced OPA1 hyperacetylation induced by LP diet during pregnancy (Figure 4b,e).

### 3.7. NR Supplementation Reduces Hyperacetylation of the SIRT3 Target SOD2 in Maternal LP Diet Offspring

**Figure 5:**
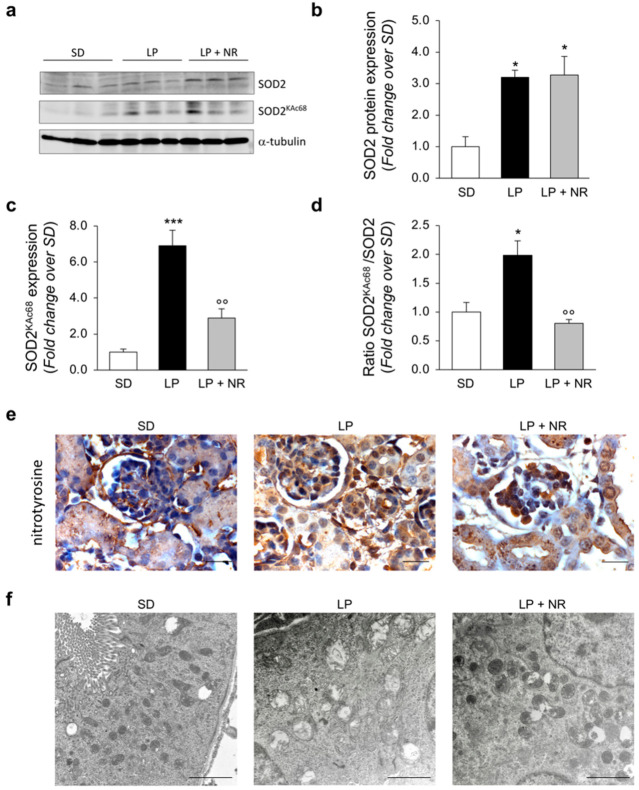
NR supplementation protects from oxidative stress by regulating SOD2 in maternal LP diet offspring, preserving mitochondrial ultrastructure. (**a**–**c**) Representative Western blots (**a**) and densitometric analysis of total SOD2 (**b**) and SOD2 acetylated at lysine 68 (SOD2^KAc68^) (**c**) in total kidney extracts harvested from newborn mice (day 1) from mothers fed a SD, LP diet alone or with NR; *n* = 3 samples per group. Each sample consisted of a pool of at least 4 kidneys from 4 newborn mice. (**d**) Densitometric analysis of SOD2 acetylation expressed as the ratio between the expression of SOD2^KAc68^ and total SOD2 in total kidney extracts harvested from newborn mice (day 1) from mothers fed a SD, LP diet alone or with NR; *n* = 3 samples per group. Each sample consisted of a pool of at least 4 kidneys from 4 newborn mice. (**e**) Representative images of nitrotyrosine signal in renal tissues from offspring from mothers fed a SD, LP diet alone or with NR. Scale bars, 20 μm. (**f**) Representative micrographs of mitochondrial alterations in proximal tubular cells of newborn mice (day 1) from mothers fed SD, LP diet alone or with NR; *n* = 3 kidneys from 3 newborn mice. Scale bars, 2000 nm. Results are presented as mean ± SEM and were analyzed with ANOVA with Tukey’s post hoc test; * *p* < 0.05, *** *p* < 0.001 vs. SD and °° *p* < 0.01 vs. LP.

We then evaluated SOD2, another target of SIRT3, whose antioxidant activity is dependent on its deacetylated status [33]. When we quantified SOD2 expression with Western Blot analysis, we found that the offspring of LP-fed mothers with or without NR had significantly higher levels of SOD2 compared to the offspring of SD-fed mothers (Figure 5a,b). Given that previous studies have identified Lysine 68 as the specific residue through which SIRT3 can regulate SOD2 activity [34], we evaluated changes in SOD2 acetylation at lysine 68 (SOD2^KAc68^). In the renal extracts from the offspring from LP-fed mothers, we found a significant increase in SOD2^KAc68^ expression compared to the offspring of SD-fed mothers, which was significantly decreased by gestational NR supplementation (Figure 5a,c). To fully assess the global SOD2 acetylation status, we normalized SOD2^KAc68^ expression to total SOD2 expression and found that NR supplementation significantly reduced SOD2 acetylation levels in renal extracts from the offspring of LP-fed mothers (Figure 5d). To study the functional relevance of modulation of SOD2 antioxidant activity in our experimental setting, we evaluated nitrotyrosine—a marker of protein oxidation in vivo—in renal tissues [18]. Immunohistochemical analysis provided evidence that newborns from LP-fed mothers exhibited an increase in the nitrotyrosine signal, while NR treatment reduced protein nitrosylation in both the glomerular and tubular compartments (Figure 5e), suggesting that NR enhances antioxidant defense in maternal LP diet offspring.

### 3.8. NR Supplementation Prevents Mitochondrial Ultrastructural Alterations in Maternal LP Diet Offspring

There is evidence that exposure to a maternal LP diet has a great impact on mitochondrial activity, including impairment of mitochondrial functions [35,36,37,38,39]. Having identified marked alterations in mitochondrial proteins in maternal LP offspring, we sought to analyze mitochondrial ultrastructure with TEM. As shown in Figure 5f, extensive damage to mitochondria was found in proximal tubular cells in the kidneys of the offspring of LP-fed mothers at day 1, as indicated by a significant increase in the number of rounded, swollen mitochondria with a loss of cristae observed in these pups, compared to the offspring of SD-fed mothers. In contrast, the renal samples harvested from the offspring of LP-fed mothers supplemented with NR exhibited a focal restoration of mitochondrial ultrastructure with preserved cristae morphology (Figure 5f). In order to evaluate the extent of mitochondrial alterations in our experimental setting, we quantified the percentage of altered mitochondria out of the total mitochondrial number. We found that the LP diet significantly increased the percentage of altered mitochondria compared to a SD (*p* < 0.001) and NR supplementation partially rescued mitochondrial ultrastructural impairment induced by LP diet (*p* < 0.05), although not to control levels (*p* < 0.01; % of altered mitochondria: SD: 7.2 ± 0.4; LP: 53.8 ± 5.5; LP + NR: 35.4 ± 2.7; mean ± SEM. Data were analyzed by ANOVA with Tukey’s post hoc test).

## 4. Discussion

Here, we provide evidence that reduced nephron endowment and impairment of the renal structure in the kidneys of mice born to mothers fed a LP diet were improved through NR supplementation. These effects were dependent on the ability of NR to normalize renal SIRT3 expression and activity, as shown by the lack of NR effectiveness in SIRT3 knockout mice. The restoration of SIRT3 by NR led to improvement in the striking alterations in mitochondrial structure induced by the LP diet.

These findings have huge potential for clinical translation, given that inadequate nephrons at birth increase the risk in adulthood of hypertension and renal diseases [4,40,41]. Chronic kidney disease affects over 10% of the adult population worldwide, with rising overall morbidity and mortality rates [42]. In view of this, it is evident that prenatal kidney care is an important global health challenge. Counteracting a nephron shortage has an additional layer of clinical relevance because nephron number in humans is set at birth and cannot be increased later in life, so prenatal intervention is the only option to modulate renal developmental programming. Our study provides evidence that, in pregnancies complicated by a protein-restricted diet, it is possible to prevent nephron shortage and reestablish the physiological glomerular structure and proper glomerular and tubular capillary network by supplementing the mother’s diet.

To modulate nephron endowment, we chose a strategy that aimed to boost SIRT3, based on our recent study, which showed that SIRT3 is a critical determinant of proper nephrogenesis and final nephron number [13]. Consistent with our study, we report the unprecedented findings that renal SIRT3 expression and activity were significantly reduced in the kidneys of mice born to mothers under gestational protein restriction regimen, which provided the rationale for targeting the SIRT3-dependent pathway to restore nephrogenesis. Among the small number of available SIRT3 activators, we focused on NR, which can increase the bioavailability of NAD^+^, the essential co-substrate for SIRT3 activity. Notably, NR takes on particular importance due to its potency in vivo and the lack of adverse clinical effects [43]. Indeed, several clinical studies are investigating the therapeutic potential of NR for treating several pathological conditions, including neurodegenerative and cardiovascular disorders [44].

In this study, we observed that SIRT3 protein expression was induced by NR treatment in the kidneys of mice born to LP-fed mothers. It has been reported that NR can activate PGC-1α [45] which, among several other important functions related to mitochondrial biogenesis and energetic metabolism [46,47], controls SIRT3 transcription through the coactivation of the orphan nuclear receptor Err (estrogen-related receptor)-α [48]. Accordingly, here we found that PGC-1α protein expression, which was significantly lower in the kidneys of LP-fed mothers’ pups, was fully restored in mice that received NR. These results clearly indicate that an NR-mediated increase in SIRT3 expression passes through an induction of PGC-1α protein expression. In addition, recent studies suggested that PGC-1α is involved in the maintenance of the NAD^+^ pool, via multiple mechanisms [49,50,51]. Despite not having evaluated the NAD^+^ content in our experimental setting, it is conceivable that NR-induced PGC-1α significantly increased the NAD^+^ levels, which are reduced by maternal malnutrition, as revealed by the altered tryptophan metabolism [52].

As a likely consequence of increased NAD^+^ bioavaibility, we documented that NR supplementation was able to induce SIRT3 activity, as shown by a significant reduction in total renal extract hyperacetylation during pregnancies complicated by dietary restriction, providing evidence of the drug’s effectiveness in activating deacetylase enzymatic activity of SIRT3. Our finding corroborates previous studies that have shown that NR treatment enhances SIRT3 activity [33,53]. It is known that SIRT3-mediated deacetylation of lysine residues of target proteins can positively regulate many intracellular pathways involved in mitochondrial fission and oxidative stress energy homeostasis and metabolism [16]. Specifically, we focused on OPA1, a protein that is important for mitochondrial integrity and the fusion of inner mitochondrial membranes, whose activity is regulated by SIRT3 through deacetylation [31]. In line with SIRT3-reduced activity, OPA1 hyperacetylation was found in the kidneys of mice born to LP-fed mothers and was decreased significantly by NR treatment. That SIRT3 is functionally impaired in the kidneys of LP-newborn mice was also confirmed by the finding of increased acetylation of SOD2, the main mitochondrial antioxidant enzyme [54]. The impairment of SOD2 activity translates into increased oxidative stress and the production of reactive oxygen species, as revealed by an increase in nitrotyrosine staining in renal tissue from mice exposed to a low protein diet during pregnancy. Notably, NR effectively enhanced SOD2, thus reducing oxidative stress in vivo, suggesting that the beneficial effects of NR on kidney development may also relate to its SIRT3-dependent antioxidant activity.

Given the profound alteration in the acetylation status of mitochondrial proteins, we investigated the mitochondrial ultrastructure. Consistent with previous studies on the liver and skeletal muscle [35,36,37,38,39], we found that exposure to a maternal LP diet had a negative impact on mitochondrial structure in the neonatal kidney. In this setting, NR significantly rescued defects in mitochondrial morphology, which is recognized as being strictly connected with key mitochondrial functions. In particular, our finding that NR was able to increase OPA1 activity via SIRT3 suggests that the restoration of mitochondrial architecture may be dependent on OPA1-mediated cristae junction organization [55]. In addition, the ability of NR to impact the activity of OPA1 plays a multifaceted role, given that OPA1 counteracts oxidative stress [56] and maintains mitochondrial permeability [57], further supporting the beneficial effect of the SIRT3/OPA1 axis on mitochondrial structure. In addition, the ability of NR to maintain SOD2 activity via SIRT3 further amplifies the beneficial effect of OPA1 on preserving mitochondrial oxidative state and function. These findings further support our previous study, which showed that intact mitochondrial functions regulated by SIRT3 are fundamental for the maintenance of a proper metabolic milieu, characterized by sustained oxidative metabolism and decreased glycolysis, to promote nephrogenesis and dictate nephron number [13]. Our findings are also consistent with previous investigations that have reported that the stimulation of NAD^+^ has protective effects on mitochondrial health across different cells in both animal models and humans [33,58,59,60,61].

## 5. Conclusions

Collectively, all our data contribute to establishing an intervention pathway to prevent nephron shortage during pregnancies that are at risk of developmental renal defects, through specific diet supplementation. Future studies should aim to investigate whether the NR-induced restoration of nephron endowment ultimately translates into increased renal resilience following additional renal insults in adult life. These findings could have significant clinical implications, given the risk of long-term deleterious consequences on the health of infants born with an inadequate nephron asset at birth and suggest that NR should be considered for clinical studies to prevent adverse programming and to support proper organ development.

## Figures and Tables

**Table 1:** Effect of NR supplementation on body and kidney weight of day 1 offspring born to mothers fed LP diet.

	SD Diet	LP Diet	LP Diet + NR
Body weight of mothers after delivery (g)	26.5 ± 0.1	25.0 ± 1.0	23.9 ± 1.3
Number of pups	5.3 ± 0.029	5.3 ± 0.029	6.0 ± 1.0
Body weight of pups (g)	1.247 ± 0.023	1.009 ± 0.003 ***	1.087 ± 0.025 ***
2 kidney weight of pups (g)	0.013 ± 0.001	0.008 ± 0.001 ***	0.012 ± 0.001 °°
2 kidney weight/body weight of pups (ratio)	1.096 ± 0.067	0.821 ± 0.060 **	1.060 ± 0.034 °

Results are presented as mean ± SEM and were analyzed with ANOVA with Tukey’s post hoc test. Mothers’ weight: *n* = 5. Body weight and kidney weight: SD, *n* = 20; LP, *n* = 15; LP + NR, *n* = 21; *** *p* < 0.001 and ** *p* < 0.01 vs. SD; °° *p* < 0.01 and ° *p* < 0.05 vs. LP.

## Data Availability

The original contributions presented in the study are included in the article. Further inquiries can be directed to the corresponding author.

## Supplementary Materials

The following supporting information can be downloaded at: https://www.mdpi.com/article/10.3390/cells11203316/s1, Figure S1: uncropped gels of representative Western Blots reported the main figures.
