# Correction: Pezzotta et al. Low Nephron Number Induced by Maternal Protein Restriction Is Prevented by Nicotinamide Riboside Supplementation Depending on Sirtuin 3 Activation. *Cells* 2022, *11*, 3316

**DOI:** 10.3390/cells14171328

**Published:** 2025-08-28

**Authors:** Anna Pezzotta, Luca Perico, Marina Morigi, Daniela Corna, Monica Locatelli, Carlamaria Zoja, Ariela Benigni, Giuseppe Remuzzi, Barbara Imberti

**Affiliations:** Istituto di Ricerche Farmacologiche Mario Negri IRCCS, Centro Anna Maria Astori, Science and Technology Park Kilometro Rosso, 24126 Bergamo, Italy

In the original publication [1], there was a mistake in Figure 3a as published. Specifically, an image from the treated group LP + NR was unintentionally positioned as a representative image of the control group SD. The authors state that the scientific conclusions are unaffected. This correction was approved by the Academic Editor. The original publication has also been updated.

**Figure 3:**
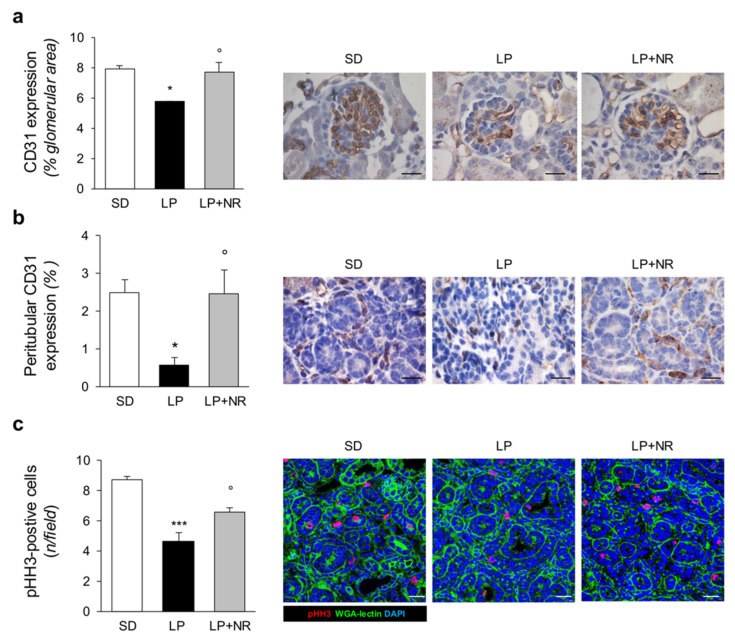
NR supplementation protects renal vasculature and increases cell proliferation in maternal LP diet offspring. (**a**) Quantification and representative images of the endothelial marker CD31 in glomeruli of SD, LP or LP + NR offspring (*n* = 3 kidneys from 3 newborn mice per group). Scale bars, 20 μm. (**b**) Quantification and representative images of the peritubular area positive for CD31 in SD, LP or LP + NR offspring (*n* = 3 kidneys from 3 newborn mice per group). Scale bars, 20 μm. (**c**) Representative images and quantification of cell proliferation assessed by phh3 staining in kidneys from day 1 mice born to mothers fed a SD or LP supplemented or not with NR (*n* = 3 kidneys from 3 newborn mice per group). Scale bars, 20 μm. Results are presented as mean ± SEM and were analyzed with ANOVA with Tukey’s post hoc test. * *p* < 0.05, *** *p* < 0.001 vs. SD; ° *p* < 0.05 vs. LP.
