# Supplementary material for: Low Nephron Number Induced by Maternal Protein Restriction Is Prevented by Nicotinamide Riboside Supplementation Depending on Sirtuin 3 Activation
Source: Cells. 2022 Oct 21;11(20):3316. doi: 10.3390/cells11203316 (PMC9600228; doi:10.3390/cells11203316)
Supplement: Supplementary file 1 [file cells-11-03316-s001.zip › cells-1954113-supplementary.pdf]

Uncropped gels in Figure 1b

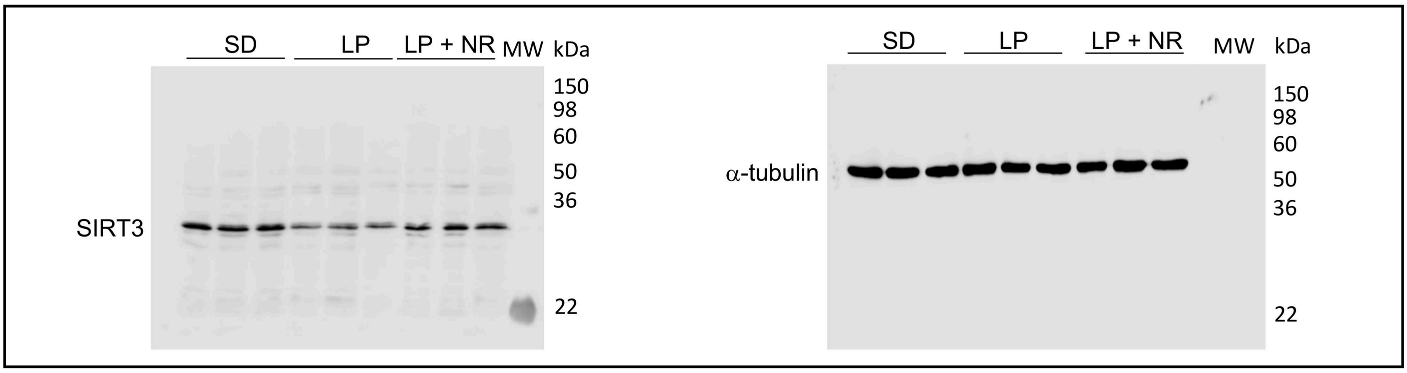

Uncropped gels in Figure 4a

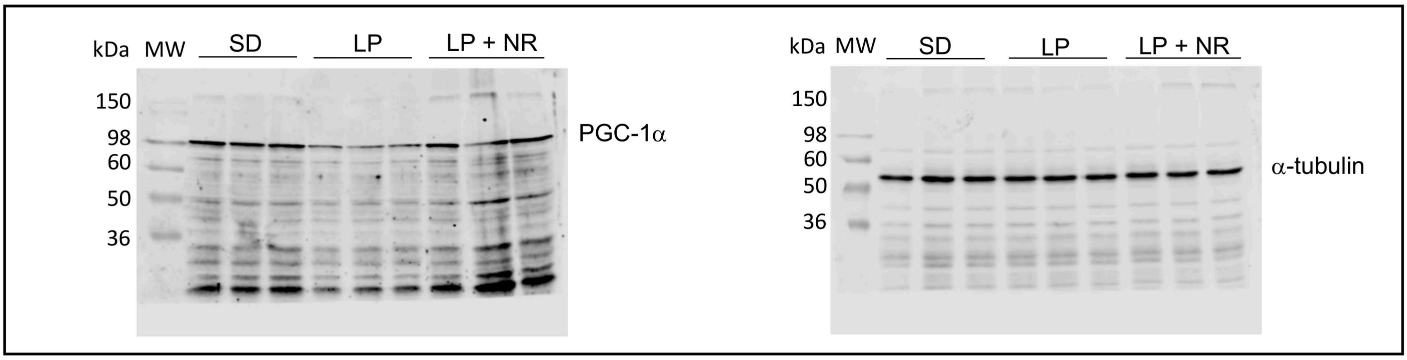

Uncropped gels in Figure 4b

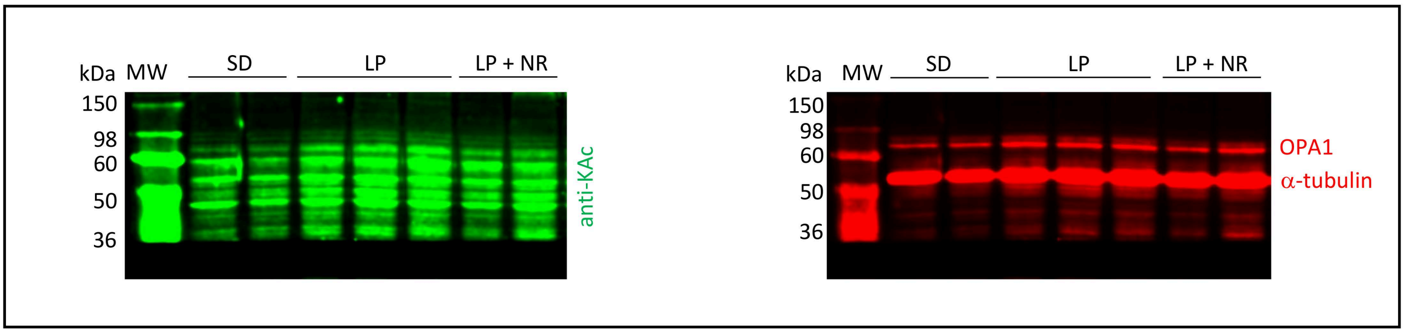

Uncropped gels in Figure 5a

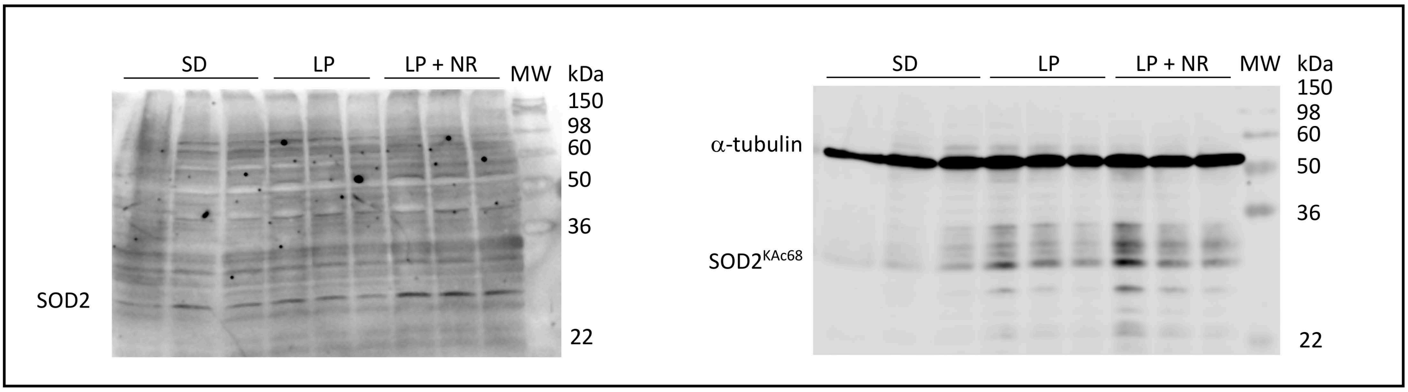

Figure S1. Uncropped gels of representative Western Blots reported the main figures. SD, standard diet; LP, low protein diet; NR, nicotinamide riboside; MW, molecular weight; kDa, kilo Dalton; SIRT3, sirtuin 3; PGC-1α, proliferator-activated receptor γ (PPARγ) coactivator-1α; KAc, lysine acetylation; OPA1, optic atrophy 1; SOD2, superoxide dismutase 2; $SOD2^{KAc68}$, superoxide dismutase 2 acetylated at lysine 68.
